# Pulmonary histopathology in fatal paraquat poisoning

**DOI:** 10.4322/acr.2021.342

**Published:** 2021-11-22

**Authors:** Senthil Kumar, Shikha Gupta, Yogender Singh Bansal, Amanjit Bal, Pulkit Rastogi, Valliappan Muthu, Vanshika Arora

**Affiliations:** 1 Postgraduate Institute of Medical Education and Research, Department of Forensic Medicine, Chandigarh, India; 2 Postgraduate Institute of Medical Education and Research, Department of Histopathology, Chandigarh, India; 3 Postgraduate Institute of Medical Education and Research, Department of Pulmonary Medicine, Chandigarh, India

**Keywords:** Paraquat, Acute toxicity, Autopsy, Histology, Lung injury

## Abstract

Paraquat is a potent herbicide widely used in the Indian agriculture industry. Human fatality due to paraquat poisoning is not uncommon in this country. The primary effect of paraquat is on the lungs, and the resultant pulmonary damage leads to the patient's demise. There is a high mortality rate in paraquat poisoning as the treatment is usually supportive with no known antidote. There are limited human studies that have observed the histopathological changes in lungs in paraquat poisoning. The authors have discussed the time-related histopathological changes in lungs in paraquat poisoning on autopsy subjects. The role of anticoagulants and fibrinolytic agents in the treatment of this poisoning has also been discussed.

## INTRODUCTION

Paraquat (1,1’-dimethyl-4,4’-bipyridylium dichloride) is a bipyridylium quaternary ammonium compound used as a potent weed killer and a herbicide in the agriculture industry. Commercially, it is sold as a brownish concentrated liquid of the dichloride salt in concentrations ranging from 10–30% under the brand name "Gramoxone" and for horticultural use as brown granules known as "Weedol" at a concentration of about 5%.^1^ Acute Paraquat poisoning, either due to accident or suicide, is a known problem in India.^2^ The most frequent route of exposure is ingestion. Exposure can occur, however, through direct skin contact or inhalation during spraying.^1^ Paraquat toxicity primarily affects the lungs because of the active, energy-dependent uptake of paraquat by the alveolar epithelium via the polyamine uptake pathway. In pneumocytes, it undergoes a redox reaction, leading to the formation of free radicals, thus causes oxidative stress.^3^ The resultant pulmonary fibrosis leads to respiratory failure and death.^4^

To our knowledge, only a few studies have been done in the past to observe the histopathological changes in lungs following paraquat ingestion or administration, and most of these studies have been done on experimental animal models. Autopsy studies in human subjects, especially on sequential lung changes, are limited.^5^^-^ 9 Herein, we describe the sequential histopathological changes of paraquat-induced lung injury identified during autopsy. We have analyzed the pattern of lung changes with respect to the survival period.

## MATERIALS AND METHODS

### Study Design and Duration:

We performed a retrospective observational study from the autopsy database of our institute from July 2018 to March 2021.

Inclusion and exclusion criteria:

Autopsy records of acute paraquat poisoning cases were retrieved and retrospectively analyzed. We included only the autopsy cases in which paraquat poisoning was clinically diagnosed based on definitive history. Acute paraquat poisoning was identified based on history and positive toxicological testing for paraquat. We excluded the cases with a history of other poison or drug ingestion along with paraquat, with other coexisting pathology or pre-existing heart or lung disease.

### Study Objectives:

1. Describe the gross and microscopic pathological findings of lungs obtained during autopsy in paraquat poisoning
2. To correlate the histopathological findings with the survival period.

We retrieved and analyzed the records, including post-mortem reports, forensic histopathology laboratory registers, slides, histopathology reports, and toxicological screening/confirmatory reports.

### Pathology Protocol:

In all selected cases, extensive sampling from all the lobes and areas from the subpleural to the central part of the lungs had already been performed. The number of lung blocks and routine hemotoxylin and eosin slides of each case varied from 8 to 15. We carried out special stains, including Masson's trichrome and Martius scarlet blue (MSB), to highlight fibroblast, collagen and fibrin deposition in the alveolar septum and spaces. Two Histopathologists and Forensic Medicine experts independently reviewed all the histopathology slides.

## RESULTS

We identified a total of 17 cases fulfilling our inclusion criteria, which were selected for the study. The gross findings varied from congestion, edema, hemorrhagic consolidation to fibrosis. The longer the survival, the more solid and fibrotic the lung was. The combined weight of lungs ranged from 924 g to 1466 g [RR: 1000 g]. The following microscopical findings were observed (Table 1).

**Table 1:** Lung histopathological findings of acute fatal paraquat poisoning.

**Lung histopathology findings observed in our case series of acute fatal paraquat toxicity**
**Pulmonary edema (15/17)^ǂ^**
**Septal congestion (16/17) ^ǂ^**
**Pulmonary hemorrhage (16/17) ^ǂ^**
**Necrosis and denudation of type I pneumocytes (5/17) ^ǂ^**
**Intra-alveolar hemosiderin laden macrophages (5/17) ^ǂ^**
**Intra-alveolar infiltration of neutrophils/lymphocytes (10/17) ^ǂ^**
**Hyaline membrane formation/Diffuse alveolar damage [DAD] (9/17) ^ǂ^**
**Intra-alveolar fibrin deposition (6/17) ^ǂ^**
**Intra-alveolar fibroblast proliferation/ collagen deposition [Masson’s body formation] (3/17) ^ǂ^**
**Proliferation of type II pneumocytes (4/17) ^ǂ^**
**Pulmonary artery thrombosis (3/17) ^ǂ^**
**Honeycomb appearance (7/17) ^ǂ^**

ǂ *Frequency of the observed alterations*

The data regarding the dose and blood level of paraquat was not available. However, as the primary aim of this paper was to correlate the histopathological findings with the survival period, we arranged the cases in ascending order based on the survival time, and corresponding prominent histopathological findings were listed (Table 2). The survival period of 17 cases ranged from 1 day to 14 days.

**Table 2:** Lung histology of acute fatal paraquat poisoning in relation with survival time.

**Case#**	**ST**	**P.E**	**S.C**	**P.H**	**HLM**	**N/L**	**HM**	**I.A.F**	**I.A.F.C**	**H.C**
**13**	1	+	+++	-	-	-	-	-	-	-
**14**	2	++	++	++	-	-	-	-	-	-
**3**	3	++	+++	+	-	-	-	-	-	-
**16**	3	+++	+++	+	+	N	-	-	-	-
**17**	3	+	++++	++++	-	N	-	-	-	-
**15**	3	+++	++++	++++	+	-	-	-	-	-
**9**	3	++	+++	++	+	N	-	-	-	-
**2**	4	++	++	++	-	N	+	-	-	-
**6**	4	+	++++	++++	-	N	++	+	-	-
**10**	4	++++	+++	+	++	N	-	-	-	+
**12**	6	+++	+	+	+	-	++	-	-	++
**1**	7	+	++	++++	-	-	+	-	-	-
**7**	7	++	++	+	-	L	+++	+	+	++
**11**	8	+	-	+++	-	-	++	+	-	++
**5**	9	-	++	+++	-	L	+++	+++	-	++
**8**	11	++++	++++	+	-	L	++	++	+	++
**4**	14	**-**	+	+	-	L	++	++	+++	+++

P.E= pulmonary edema; S.C= septal congestion; P.H= pulmonary hemorrhage; HLM= hemosiderin laden macrophages; N/L= neutrophils/lymphocytes inflamation; HM= hyaline membrane; I.A.F= Intra-alveolar fibrin; I.A.F.C = intra-alveolar fibroblast and collagen; H.C= honeycombing. ST=survival time ( days).

**Figure 1:**
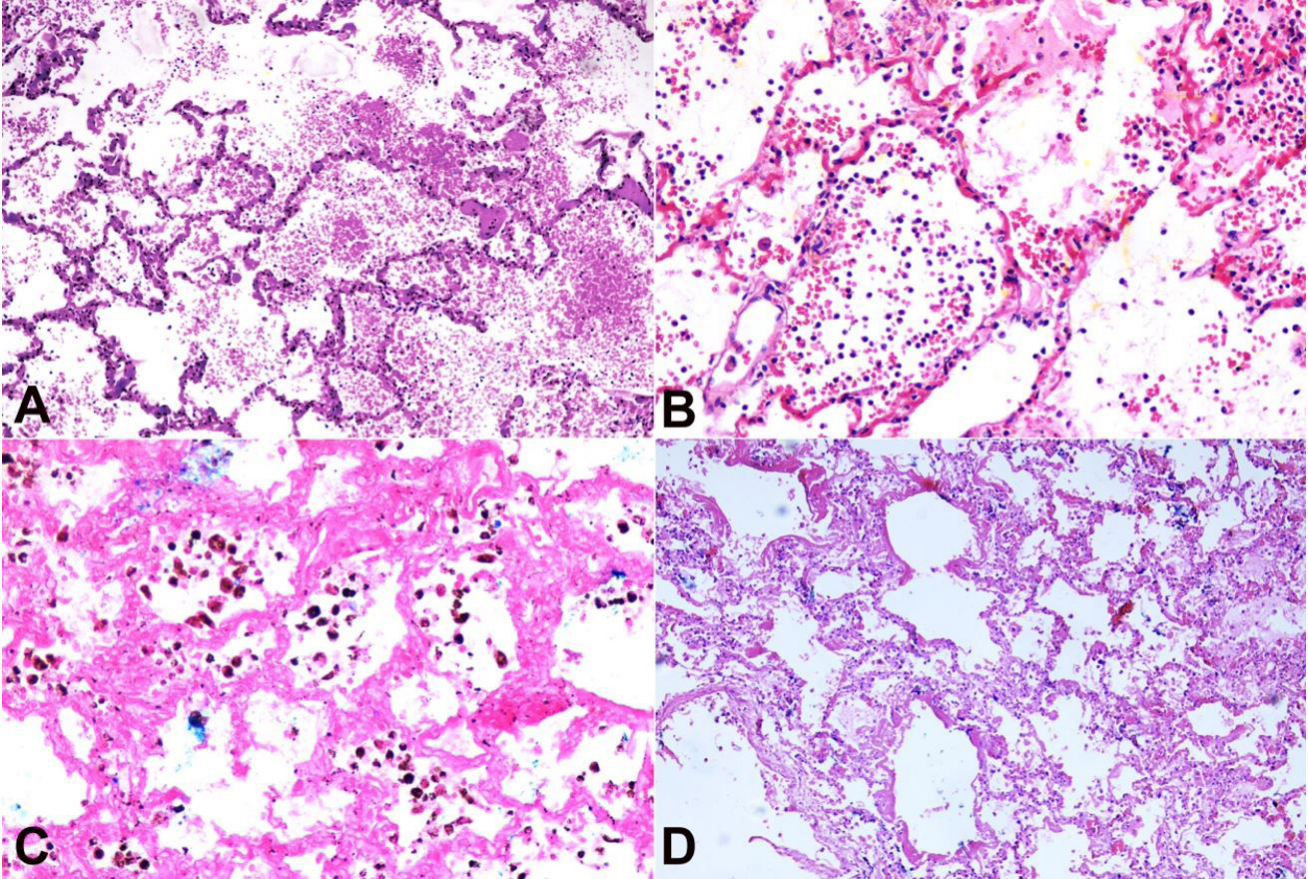
Photomicrograph of lungs: **A** – showing septal congestion with intra-alveolar hemorrhage (H&E 10X); **B** – showing septal congestion, intra-alveolar hemorrhage, and infiltration of neutrophils (H&E 20X); **C** – hemosiderin-laden macrophages in intra-alveolar spaces (Perl's Prussian blue 20X); D- hyaline membrane in alveolar walls (H&E 10X).

Pulmonary edema, septal congestion, and pulmonary hemorrhage were the most prominent findings in our study, and they were present irrespective of the survival period (Table 2). Pulmonary edema was observed in 15 (15/17) cases, septal congestion and hemorrhage were seen in 16 (16/17) cases. Ten cases showed inflammatory infiltrates in alveolar spaces; among those cases, neutrophilic infiltration was seen in six cases of survival period 3 to 4 days. Lymphocytes were seen in the rest of the four cases of survival period ranged from 7 to 14 days. Five cases of survival period between 3 and 6 days showed hemosiderin-laden macrophages in the alveolar spaces (Figure 1).

**Figure 2:**
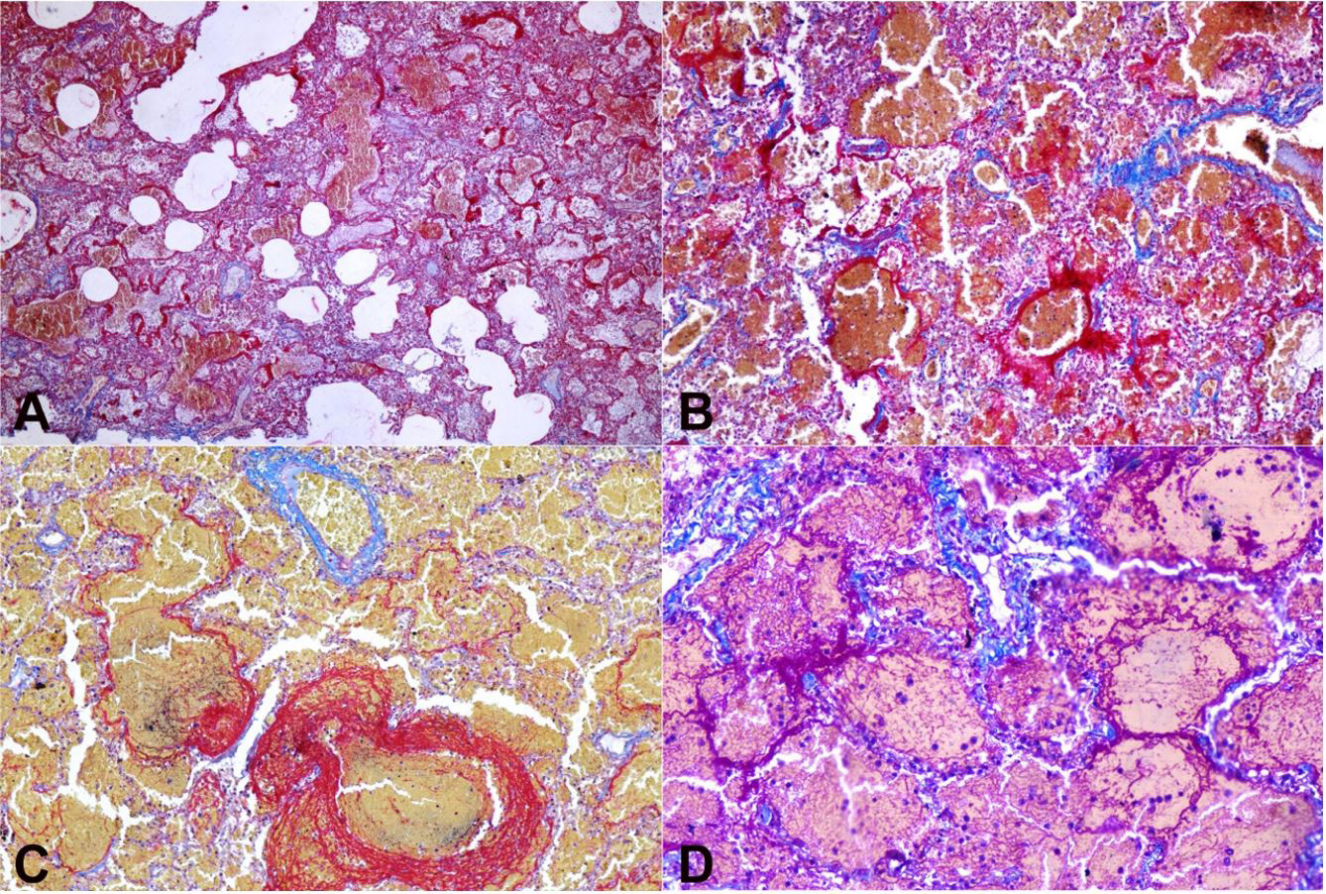
Photomicrographs of lungs: **A, B** – Hyaline membrane highlighted with Martius scarlet blue in bright red (M.S.B 4X,10X); **C** – Hyaline membrane and intra-alveolar fibrin deposition highlighted by M.S.B (20X); **D** – Hyaline membrane and intra-alveolar fibrin deposition highlighted by Masson's trichrome stain (20X).

The phagocytes were not constant, and they tended to disappear with time. The other striking finding was fibrin deposition along the alveolar wall (hyaline membrane) and in the alveolar space. A total of 9 cases (9/17) showed a hyaline membrane, which was highlighted by MSB stain (Figure 2). The earliest appearance of the hyaline membrane was observed on the 4^th^ day and seen till the 14^th^ day. Intra-alveolar fibrin deposition was observed in 6 cases, the earliest appearance was on 4^th^ day, and it was consistent and more prominent from 7^th^ day.

**Figure 3:**
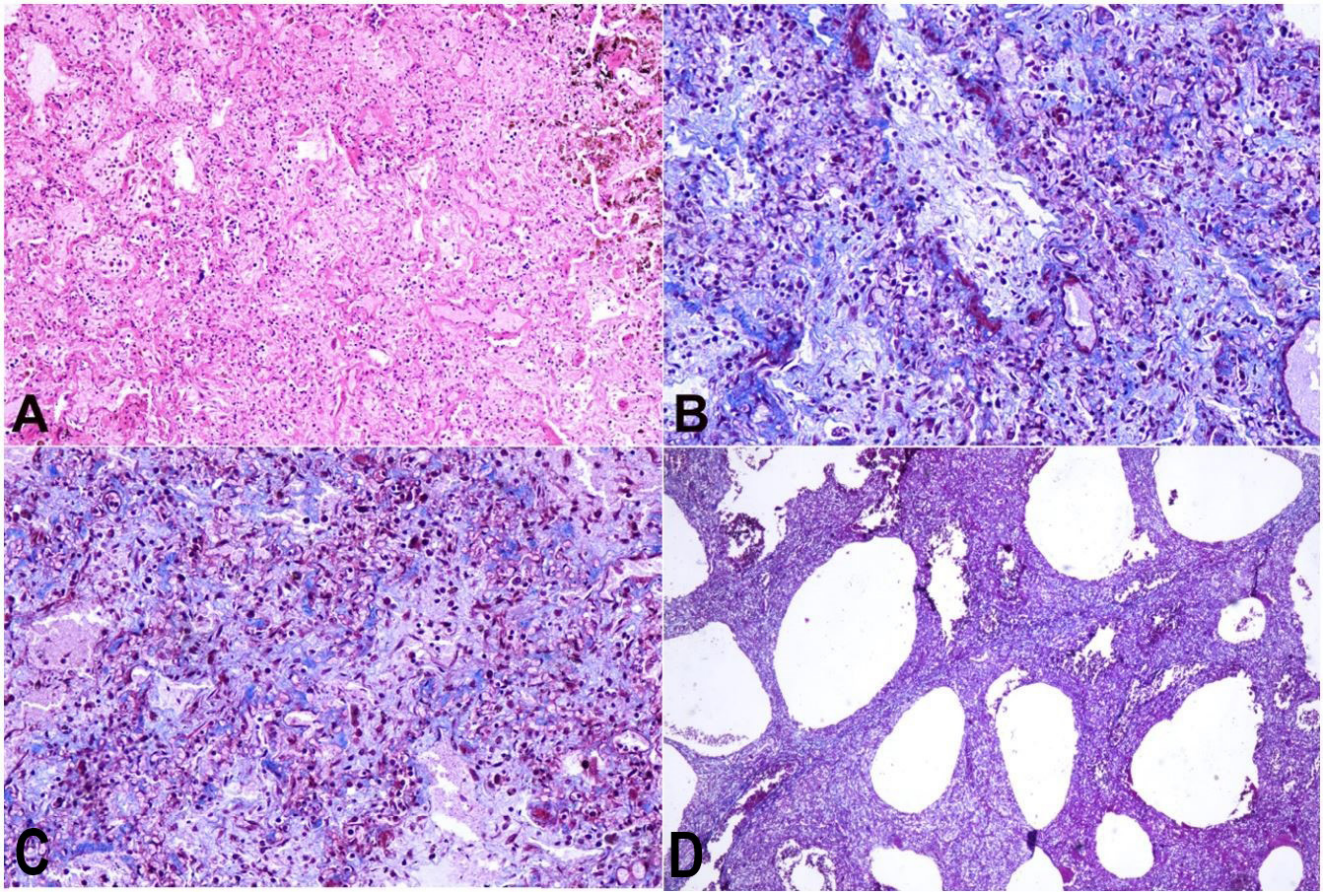
Photomicrographs of lungs: **A** – showing fibroblast infiltration and collagen deposition in alveolar spaces with obliteration of alveolar architecture (H&E 10X); **B, C** – showing intra-alveolar fibroblasts and collagen deposition. The collagen fibers are stained in blue color (Masson's trichrome 20X); **D** – honeycomb appearance of the lung (Masson's trichrome 20X).

Three cases with a long survival period (7 to 14 days) showed fibroblast and collagen deposition in alveolar spaces (Figure 3). Collagen deposition was extensive in the case with 14 days survival. From the 7^th^ day, there was a time-dependent increase in fibroblast proliferation and collagen deposition in the alveolar spaces. The intra-alveolar fibrosis with obliteration of alveolar architecture was extensive with the increase in time. One more consistent finding was the honeycomb appearance of the lung parenchyma due to dilatation of respiratory bronchioles. Honeycombing was prominent at subpleural areas and worsened with time due to progressive obliteration of alveolar spaces by fibrosis (Figure 3D).

## DISCUSSION

Paraquat is a bipyridilium herbicide that accumulates selectively in the lungs via a saturable uptake process. Paraquat's systemic toxicity is primarily caused by its effects on the lungs.^3^ Paraquat poisoning has a long-term clinical course, and there is no known antidote. The patient usually succumbs to respiratory failure as a result of progressive pulmonary fibrosis.^10^ The detail of the morphological changes of the pulmonary lesions in paraquat toxicity is a point of contention. There are often diverging interpretations of the morphological changes induced by paraquat poisoning due to the differences in experimental design, species used, the dose and route of paraquat administration, and the time interval of exposure.^11^ We attempted a study on sequential morphological changes of lungs in fatal paraquat poisoning cases. As there is no promising treatment to prevent paraquat-induced lung injury, studying the exact disease process would help find a therapeutic intervention to reduce the paraquat fatality.

Paraquat, by its oxidation-reduction reaction, interferes with the mitochondrial respiratory chain, leading to the formation of oxygen free radicals. These free radicals cause lipid peroxidation and damage the endothelial cells of alveolar capillaries and pneumocytes.^12^ An animal study by Dearden LC et al.^13^ showed electron microscopic evidence of alveolar epithelial injury 24 hours post paraquat, and the damage was maximum at 72-96 hours. It also showed evidence of endothelial damage that started at 48 hours and was maximum at 72 to 96 h post-paraquat. The endothelial damage leads to capillary congestion and leakage of red blood cells and other inflammatory cells into alveolar spaces. Pulmonary capillary damage along with alveolar epithelial damage was proposed to be the cause of pulmonary edema. Gardiner^14^ has proposed damage to the type 2 pneumocytes causes decreased secretion of surfactant, leading to increased intra-alveolar fluid surface tension. This results in the withdrawal of fluid into alveolar spaces from capillaries. In our case series, we found septal capillary congestion, interalveolar edema and hemorrhage as predominant findings irrespective of the survival period. The majority of cases with survival period of 3 to 4 days showed intra-alveolar infiltration of neutrophils. These findings are in favour of paraquat-induced damage to the alveolar epithelium and alveolar capillary endothelial cells.

The role of extravascular fibrin deposition in acute lung injury (ALI) is well established. Intra alveolar fibrin deposition, intravascular thrombosis or disseminated intravascular coagulation are notable features of diverse forms of ALI.^15^ Our findings strongly establish the role of activation of the local coagulation cascade in paraquat-induced acute lung injury. In paraquat toxicity, pneumocyte and endothelial cell injury would result in overexposure of local tissue factor and subsequently activation of coagulation cascade and deposition of fibrin along the alveolar wall and also in alveolar spaces. The fibrin polymers deposited along the alveolar wall forms the hyaline membrane. All these changes are seen in the destructive phase or exudative phase of paraquat toxicity.^8^ There is enough evidence to suggest that fibrin deposition in alveoli in the early exudative phase of ALI may further progress into fibrosis by organization and remodeling in the later stage. In our case series, fibrin deposition along septum and intra-alveolar spaces started on the 4^th^ day and was more prominent from the 7^th^ day. Subsequently, intra-alveolar fibroblast and collagen appeared from the seventh day, and the collagen deposition and obliteration of alveolar architecture became more prominent as time increases. This later stage of ALI is called the proliferative phase, and honeycombing due to dilatation of respiratory bronchioles is also a prominent finding.

To summarize the sequential changes of paraquat-induced ALI, our findings can broadly fit into two phases; exudative and proliferative phase.

### Exudative Phase:

1. Damage to the alveolar-capillary barrier (endothelium and alveolar epithelial cells) leads to its increased permeability results in septal congestion, intra-alveolar edema, hemorrhage and neutrophil Infiltration.
2. Fibrin deposition along the septal wall where the epithelium is denuded (Hyaline membrane/DAD) and in the alveolar spaces.

### Proliferative Phase:

1. Intra-alveolar fibroblast proliferation/collagen deposition indicating organization of hyaline membrane
2. Occasionally hyaline membrane organizes by intra-alveolar fibroblastic proliferation (Masson's body formation)
3. Obliteration of alveolar spaces by lung fibrosis

The treatment options for paraquat poisoning is limited. In fact, the only proven treatment at present is low tidal volume lung-protective mechanical ventilation.^16^ The routine use of antioxidants and other conservative therapy is not proved successful, and the mortality rate is 90%.^17^ Considering the primary pathology, i.e., fibrin deposition consequent to the alveolar-capillary barrier damage, antithrombin therapy may be helpful in paraquat-induced ALI. Thrombin (Factor IIa) is a highly active serum protease that acts through protease-activated receptors(PARs).^18^ PARs are present in many cell types, including lung alveolar epithelium^19^ and neutrophils.^20^ Blockage of this receptor reduces fibrin deposition neutrophil influx.^21^^,^ 22 Regarding the route of administration, experimental models had demonstrated differential responses to lung injury when treatment was administered directly to air space compared with systemic treatment. In a sheep model of pneumonia and smoke inhalation induced lung injury, inhaled heparin significantly reduced lung vascular leak compared to systemic heparin administration.^23^ Montoya Giraldo et al.^24^ reported a case of severe paraquat toxicity unexpectedly survived due to the addition of enoxapene, a low molecular weight heparin with the standard treatment. From these findings, we hypothesize that inhalation antithrombin therapy in the exudative phase of acute paraquat poisoning may reduce lung vascular leakage and fibrin deposition. It may also prevent the progression of ALI to pulmonary fibrosis. This hypothesis should be extensively studied in animal models of paraquat-induced lung injury and by further clinical trials.

## CONCLUSION

Our findings strongly suggest that fibrin deposition induced by pneumocyte and alveolar-capillary endothelium damage is the major pathology in acute paraquat toxicity. We observed diffuse alveolar damage in the early exudative phase and fibroblast proliferation and collagen deposition in alveolar spaces in the late proliferative phase. Considering the pathology, anticoagulants and fibrinolytic interventions may attenuate paraquat-induced lung injury and decrease mortality. The hypothesis that the reversal of abnormal fibrin turnover in paraquat-induced ALI would prevent lung damage is worth to be tested. Furthermore, a study of the effects of such treatment in the early and late stages of paraquat poisoning is required.
